# Imaging 2-hydroxyglutarate and other brain oncometabolites pertinent to critical genomic alterations in brain tumors

**DOI:** 10.1259/bjro.20210070

**Published:** 2023-03-22

**Authors:** Teena Thomas, Sunitha Thakur, Robert Young

**Affiliations:** 1 Departments of Radiology, Memorial Sloan Kettering Cancer Center, New York, United States; 2 Medical Physics, Memorial Sloan Kettering Cancer Center, New York, United States; 3 The Brain Tumor Center, Memorial Sloan Kettering Cancer Center, New York, United States

## Abstract

The 2021 World Health Organization (WHO) Classification of Tumors of the Central Nervous System (CNS) and recent smaller annual updates have shown that alterations in tumor genetics are essential to determining tumor diagnosis, biological activity, and potential treatment options. This review summarizes the most important mutations and oncometabolites, with a focus on the central role played by 2-hydroxyglutarate in isocitrate dehydrogenase mutant tumors, as well as their corresponding imaging counterparts using standard and advanced imaging techniques.

## Introduction

Advances in technology have facilitated our understanding of many cancers through the discovery of cancer-associated genetic alterations that encode key metabolic enzymes. Specific genetic alterations may result in the accumulation of particular oncometabolites that may then drive or reflect malignant transformation, tumor evolution, and propagation. Oncometabolites can be utilized clinically to aid in diagnosis, decision making, cancer management, and prognosis. They can also be used as targets for drug and other treatment options.^1^

Perhaps the most profound application of genetic alterations was their addition into central defining roles in the 2016 World Health Organization (WHO) Classification of Tumors of the Central Nervous System. For the first time, the classification added molecular features to supplement and sometimes supplant histology in defining specific tumors. This diverged from the 2007 WHO, as well as earlier classifications that relied solely on microscopic similarities based on the cells of origin and level of differentiation. This reorganization improved discrimination between similar tumors and aligned better with clinical and biologic disease courses, while improving diagnostic accuracy, prognosis prediction, treatment decisions, and patient management.^2^ The role of isocitrate dehydrogenase (IDH) status was central to the reorganization. In this review, we will focus on imaging 2HG as an oncometabolite and its clinical applications in patients with IDH-mutant gliomas. We also discuss other oncometabolites and updates from the 2021 WHO Classification of Tumors of the Central Nervous System.

## Idh status as a defining mutation in gliomas

Determining canonical IDH status allowed the 2016 WHO Classification of Tumors of the Central Nervous System to resolve the overlapping features between tumors of astrocytic origin and tumors of oligodendroglial origin. Beyond IDH status, were 1p19q and other genetic parameters. These led to the major categories of IDH-mutant 1p19q intact astrocytoma, which is usually also ATRX-mutant and TP53-mutant, versus IDH-mutant 1p19q codeleted oligodendrogliomas. In cases where the use of both histology and molecular features result in inconsistent results, the genotype will take precedence,^2^ and it is therefore possible to classify tumors even when lacking classic histologic features. For example, a tumor with astrocytic features but IDH mutation and 1p19q codeletion will be definitively classified as an IDH-mutant 1p19q codeleted oligodendroglioma. The 2016 WHO Classification therefore provided clarity and nearly eliminated the previous mixed “oligoastrocytoma” category. Astrocytomas and oligodendrogliomas could be either Grade 2 or Grade 3. The most malignant category of WHO Grade four included both IDH-mutant glioblastoma and IDH-wildtype glioblastoma, which was problematic as the IDH-mutant glioblastomas were “secondary” and usually transformed from lower grade gliomas with better outcomes than the IDH-wildtype “primary” glioblastomas that arose *de novo*.

This and other inconsistencies have been addressed by annual cIMPACT-NOW (the Consortium to Inform Molecular and Practical Approaches to CNS Tumor Taxonomy) updates to the 2016 WHO Classification. To improve risk stratification for patients with Grade 2 and 3 IDH-mutant astrocytomas, the cIMPACT-NOW updates five investigated potential morphologic, proliferative, or molecular markers correlated with aggressive clinical behavior to incorporate into an updated grading scheme. The homozygous deletion of *CDKN2A/B* was recognized as a marker of poor prognosis independent of WHO grade, and therefore added as a criterion for “WHO Grade 4” tumor.^3^ IDH-mutant astrocytomas thereby consisted of Grade two tumors (well-differentiated; no anaplasia; absent or low mitoses; absent microvascular proliferation, necrosis, and *CDKN2A* homozygous deletion), Grade three tumors (focal or dispersed anaplasia; high mitoses; absent vascular proliferation, necrosis, and *CDKN2A* homozygous deletion), and Grade four tumors (microvascular proliferation; necrosis; or *CDKN2A/B* homozygous deletion). To unify terminology, recognize their similarity to IDH-mutant WHO Grade 2 and 3 astrocytomas, and add grading within glioma types, the 2021 WHO Classification eliminated the old “glioblastoma IDH-mutant WHO Grade 4” and replaced it with “astrocytoma IDH-mutant WHO Grade 4.”^2^

The IDH-wildtype gliomas were a focus of the cIMPACT-NOW Updates 3 and 6.^4,5^ Update three had suggested the term “diffuse astrocytic glioma, IDH-wildtype, with molecular features of glioblastoma, WHO grade IV” for IDH-wildtype gliomas with any one of these: *TERT* promoter mutation or *EGFR* gene amplification or chromosome seven gain with chromosome 10 loss (+7/–10). Recognizing that survival was similar to patients with histologically classic glioblastoma IDH-wildtype WHO Grade 4, however, the cIMPACT update six recommends the diagnosis of “glioblastoma, IDH-wildtype, WHO Grade 4” for tumors with microvascular proliferation or necrosis or any of the molecular features of glioblastoma (TERT promoter mutation or EGFR gene amplification or +7/–10 chromosome changes).^5^ The 2021 WHO Classification further defined glioblastoma as exclusively IDH-wildtype.^2^

### Central role of IDH

Underlying the rapidity and foundational changes in the 2016 WHO Classification and cIMPACT-NOW updates is the central role of IDH status. IDH mutations are the most common genomic aberration, occurring in nearly 80% of lower grade gliomas.^6^ IDH mutations are not unique to gliomas, however, as they may also be found in many other cancers, such as cholangiocarcinoma, acute myeloid leukemia, melanoma, and uterine corpus endometrial carcinoma.^7^ IDH mutation is thought to be an early driving event in the development of astrocytomas and oligodendrogliomas. There have been no cases in which IDH1 mutation occurred after a TP53 mutation or loss of 1p19q event, suggesting that astrocytomas and oligodendrogliomas may derive from a common glial precursor cell population carrying IDH1 mutations. The subsequent gain of TP53 mutations or loss of 1p19q further downstream establishes astrocytic and oligodendroglial phenotypes, respectively.^8^ The most common mutation occurs at a single amino acid residue of IDH1, arginine 132, which is commonly mutated to histidine (R132H).^9^ The IDH2 mutations, although less common than IDH1 mutations, occur at arginine 172 (R172).^10^ The IDH1 and IDH2 mutations are mutually exclusive, in that a tumor will only have one of these two mutations.

### IDH mutations result in the accumulation of 2hg

**Figure 1.**
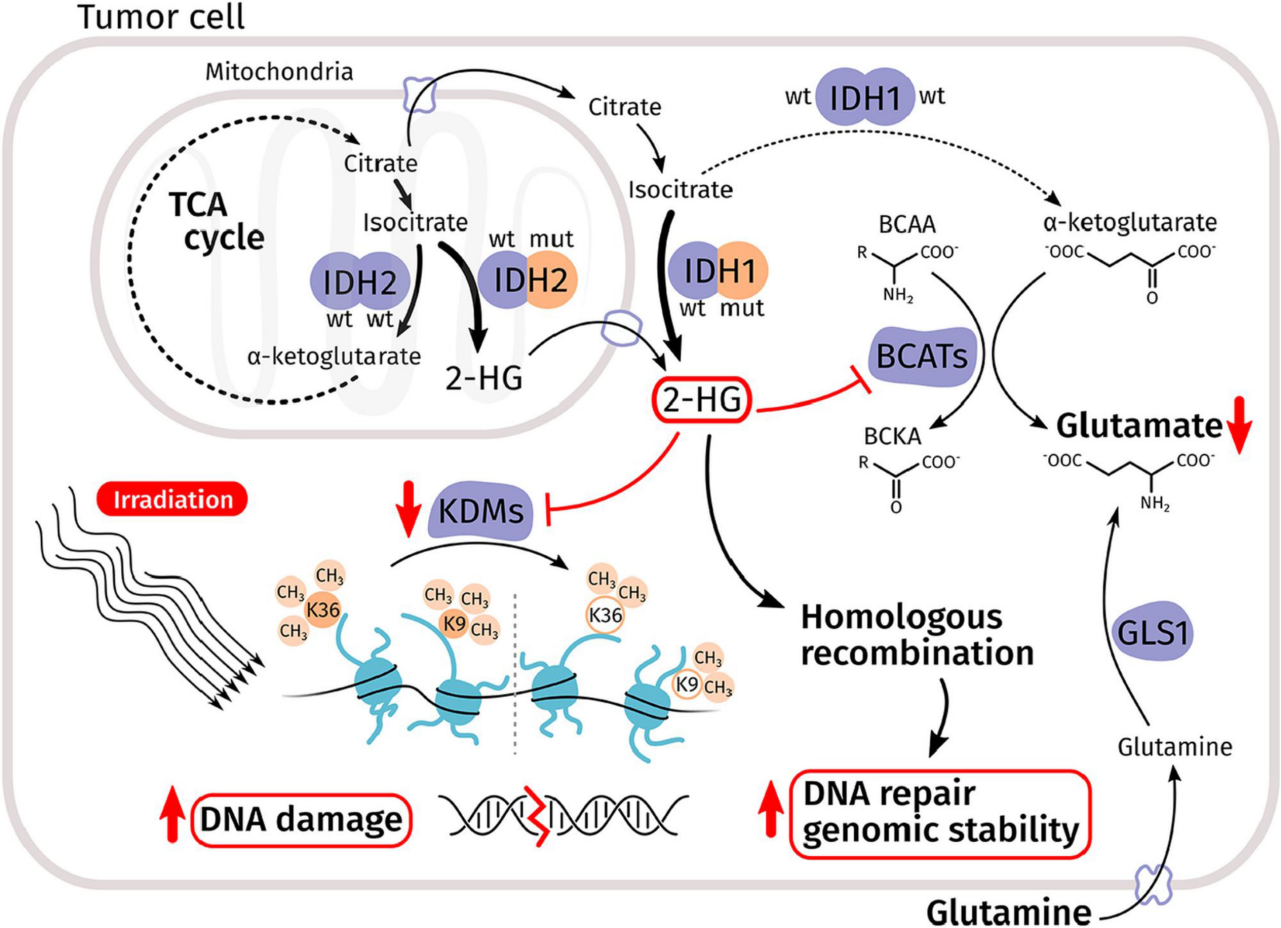
The citric acid (TCA) cycle. Oncogenic mutations in cytoplasmic or mitochondrial isocitrate dehydrogenase (IDH) result in the gain-of-function and production of the 2- hydroxyglutarate (2HG) oncometabolite that interferes with α-ketoglutarate- dependent enzymatic functions. Inhibition of branched chain amino acid aminotransferases decreases glutamate production and enhances cell reliance on glutaminase for glutathione synthesis. Inhibition of lysine histone demethylases can compromise DNA damage repair, and in other cases promote homologous recombination and genomic stability. Borrowed from.^12^

The IDH1 (cytosol) and IDH2 (mitochondrial) enzymes normally function in the citric acid (TCA) cycle to catalyze the oxidative decarboxylation of isocitrate to α-ketoglutarate.^11^ Mutation of a single gene copy is sufficient to confer a unique gain-of-function mutation, which further catalyzes the NADPH-dependent reduction of α-ketoglutarate to the R-2-hydroxyglutarate (2HG), resulting in reduced α-ketoglutarate and accumulation of excess 2HG. Figure 1. Excess 2HG can contribute to tumorigenesis by increasing the level of reactive oxygen species and competitively inhibiting glutamate and/or α-ketoglutarate-dependent dioxygenases, such as the histone demethylases necessary for progenitor cell differentiation into terminally differentiated cells, and the TET family of 5-methylcytosine hydroxylases.^13–15^ The direct action of mutant IDH1 on α-ketoglutarate may explain the predominance of IDH1 mutations in tumors originating from CNS tissue, which uniquely have high levels of glutamate uptake that are readily converted to α-ketoglutarate in the cytosol, thereby providing high levels of substrate for 2HG production.^11^ The IDH-mutant gliomas have a distinctly better prognosis than the IDH-wildtype gliomas, with further refinement of this difference possible by also considering 1p19q codeletions (median survival 6.3 years if 1p19q intact and 8.0 years if codeletion), as well as TERT and CDKN2A/B alterations.^16–18^

As an abnormally accumulating metabolite in association with specific gene mutations in the key metabolic enzyme IDH, 2HG is a prototypical oncometabolite that both plays a central role in gliomagenesis and presents unique opportunities for imaging. Accumulation of 2HG to high levels in tumors can cause metabolic and non-metabolic dysregulation and malignant transformation.^15^ Unlike IDH-mutant leukemias whose increased 2HG may be found in the blood, IDH-mutant gliomas have normal blood levels and efforts to detect 2HG have thus far centered on imaging predominantly with proton magnetic resonance spectroscopy (MRS).

## Imaging for Idh status

### MRI spectroscopy to detect and quantify 2hg

**Figure 2.**
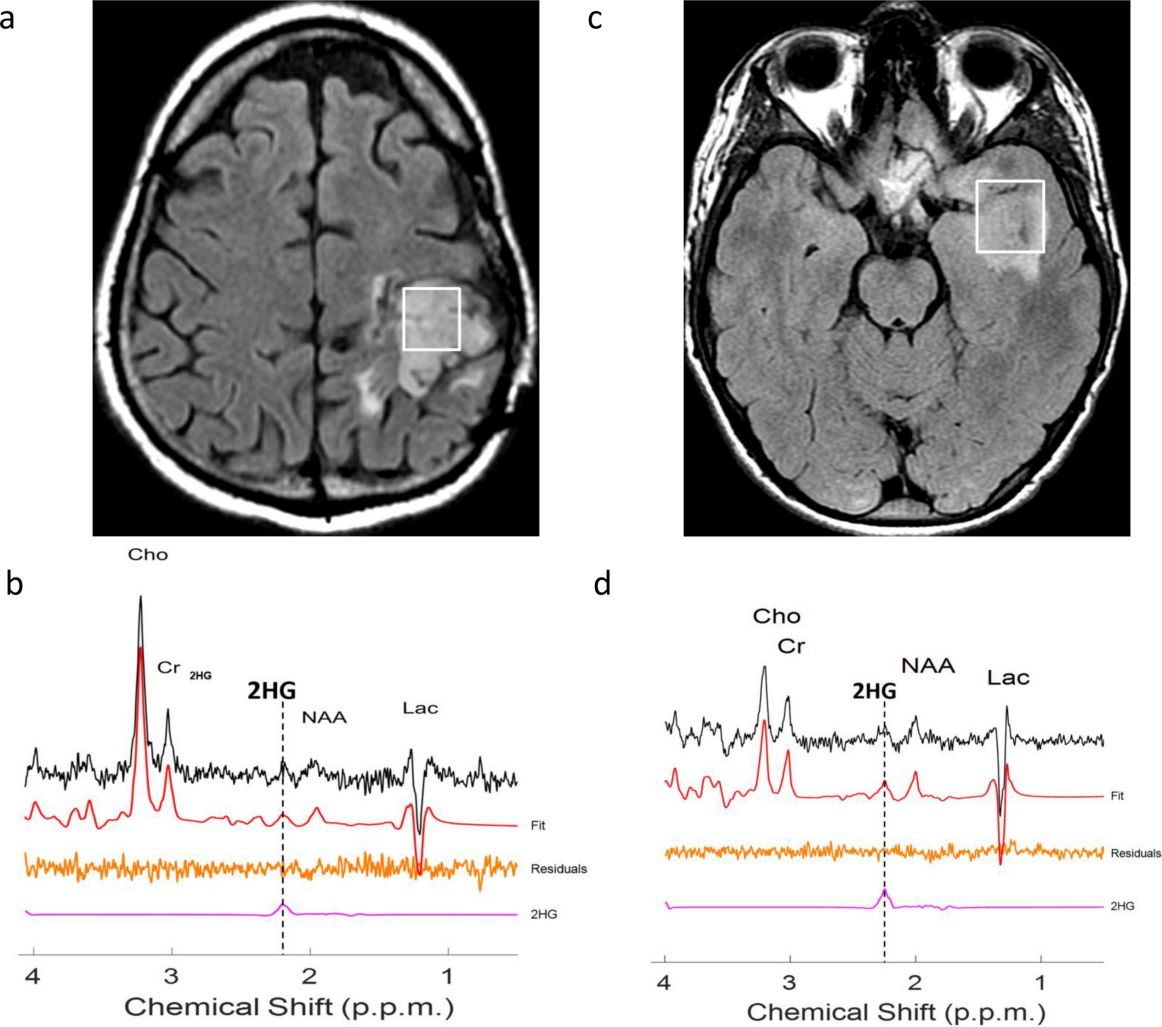
Greater increases in 2HG in IDH2-mutant gliomas. Axial FLAIR (A,C) and 2HG spectroscopy with TE=97ms (B,D) in two patients. The patient with the tumor centered in the left parietal lobe (A,B) has an IDH1 R132H-mutant glioma with increased 2HG (6.2 mM, 18% CRLB). The patient with the tumor centered in the left temporal lobe (C,D) has an IDH2-mutant glioma with more markedly increased 2HG (11.9 mM, 10% CRLB). CRLB=Cramer-Rao lower bound, an estimate of the lower bound of the variance of unbiased estimator.

MRS is an imaging technique that analyzes the chemical composition of tissues and measures metabolite concentrations in a quantitative or semi-quantitative manner. The MRS therefore provides unique functional information to complement standard MRI demonstrating anatomy.^16^ The mutant IDH enzyme confers a gain-of-function to further convert α-ketoglutarate into 2HG, leading to up to 10x increases in 2HG in IDH-mutant tumor cells against minimal background levels of 2HG in IDH-wildtype tumor cells and normal cells.^19^ The 2HG oncometabolite has five non-exchangeable, J-coupled proton resonances that may be measured using widely-available clinical 3T MRI scanners. This presents a unique opportunity for MRS to noninvasively measure increased 2HG levels *in vivo,* although specialized acquisition and analysis techniques are necessary to resolve the 2HG peak(s). Most MRS sequences have targeted the C4 proton resonances at ~2.25 ppm by optimizing point-resolved spectroscopy (PRESS) techniques.^19–23^ Single-voxel MRS PRESS with TE = 97 ms with a larger voxel (2 × 2 x 2 cm^3^) has shown greater accuracy compared to TE = 30 ms.^24^ A subsequent study established quantitative evaluation of 2HG in IDH-mutant gliomas by MRS to be both reproducible and to reliably reflect the clinical change in disease from an indolent stage through post-treatment. MRS suggests different levels of functionality between IDH mutations, with the IDH2-mutant gliomas demonstrating greater (>3x) increases in 2HG than IDH1-mutant gliomas.^25^ Figure 2.

These differences may reflect greater oxidative phosphorylation rather than aerobic glycolysis in IDH2-mutant gliomas from IDH-included metabolic reprogramming.^25^ Increases in 2HG detected by MRS are also dependent upon glioma grade, but not histologic subtype, and are strongly correlated to tumor cellularity. The highest 2HG levels were found in higher grade gliomas with high tumor cellularity. Lower 2HG levels may be found in lower grade gliomas and with low tumor cellularity regardless of glioma grade.^11,26^ Transformation from low-grade to high-grade glioma is associated with significantly increased tumor volume, a marked increase in tumor cellularity and MIB-1 proliferation index, as well as up to three-fold increase in 2HG levels. Effective treatment with IDH inhibitors, cytotoxic therapy, or vaccine therapy should result in decreased 2HG levels,^26,27^ with more rapid decreases in oligodendrogliomas than in astrocytomas, possibly related to their increased chemosensitivity.^26,28^ The 2HG MRS is therefore a promising noninvasive metabolic imaging biomarker that can aid the imaging diagnosis of IDH-mutant gliomas versus IDH-wildtype gliomas and other non-glioma tumors, as well as inform clinical management decisions during treatment.

Future 2HG MRS single voxel and single slice techniques may involve semi-localization by adiabatic selective refocusing (sLASER) and J-difference MEsher-GArwood-semi-LASER (MEGA-sLASER) techniques. These are more resilient to field inhomogeneities and more sensitive to smaller amounts of 2HG with greater fit reliability measured as lower Cramer-Rao lower bound (CRLB) or estimate of the lower bound of the variance of unbiased estimator.^29^ 2D correlation spectroscopy (2D-COSY) separates overlapping metabolite peaks by dispersing multiplet scalar J-coupled spine systems into a second spectral dimension. The pulse sequences use preparation time, evolution period, mixing time, and detection period to record signal as the function of two-time variables: t1 incremented time delay and t2 fixed time delay.^30^ 3D spectroscopy techniques, such as 3D PRESS, echo-planar acquisition with spin-echo excitation (3D-EPSI), and functional spectroscopic mapping (fSM) allow for whole tumor and/or whole brain coverage to fully interrogate irregular and potentially heterogeneous tumors, which should then improve imaging monitoring during treatment.^31–33^ Imminent improvements may include matched accumulation and sparse sampling to both reduce scan times (currently 5–15 min) and harness the potential of 7T and higher field strengths.

### pH-sensitive and oxygen-sensitive molecular MR imaging

Molecular MR imaging that is both pH-sensitive and oxygen-sensitive also has utility in identifying IDH status. The consumption of α-ketoglutarate in IDH-mutant tumors can result in decreased α-ketoglutarate dependent prolyl hydroxylase (PHD) enzyme, which normally catalyzes the oxygen-dependent degradation of the PHD substrate hypoxia inducible factor 1 α (HIF-1α). HIF-1α is a key transcription factor involved in mediating cell energy production and promoting angiogenesis in conditions of low oxygen, which is known to have strong correlation with poor patient prognosis in cancers.^10,11,13,14^ The 2HG is thought to activate PHD to increase HIF-1α degradation, thereby resulting in continued oxidative phosphorylation and a more highly oxygenated microenvironment. In contrast, the greater HIF-1αlevels in IDH-wildtype tumors transitions glucose metabolism from oxidative phosphorylation to the less efficient glycolytic pathway and subsequently increases lactic acid that lower extracellular pH..^34,35^ These changes can be captured by multiecho amine chemical exchange saturation transfer spin-and-gradient-echo echoplanar imaging (CEST-SAGE-EPI) that is pH-sensitive by quantifying the chemical exchange between amine protons in bulk water, and by quantifying the reversible transverse relaxation rate R_2_’ when oxygen-sensitive.^36^ The magnetization transfer ratio asymmetry (MTR_asym_) at 3.0ppm, which is correlated with pH, is lower in IDH-mutant tumors, consistent with less acidic environments. The transverse relaxation rate’s R_2_’, which are correlated with oxygen extraction, are also lower in IDH-mutant tumors, consistent with less hypoxic environments.^37^

### T2-FLAIR mismatch

2HG MRS is the preferred and most direct way to image the 2HG oncometabolite product of the mutant IDH enzyme, with higher sensitivity and specificity than other signs that include younger age, frontal lobe location, less contrast enhancement, well-circumscribed margins, high apparent diffusion coefficient (ADC), and lower perfusion.^38,39^ There are significant challenges, however, to broad 2HG MRS availability, including requiring local expertise, 3T or higher field strength MRI scanners, ideal target size>2 cm^3^, non-standard acquisition parameters, and difficult analysis due to spectral overlap with amino acids and other metabolites.

**Figure 3.**
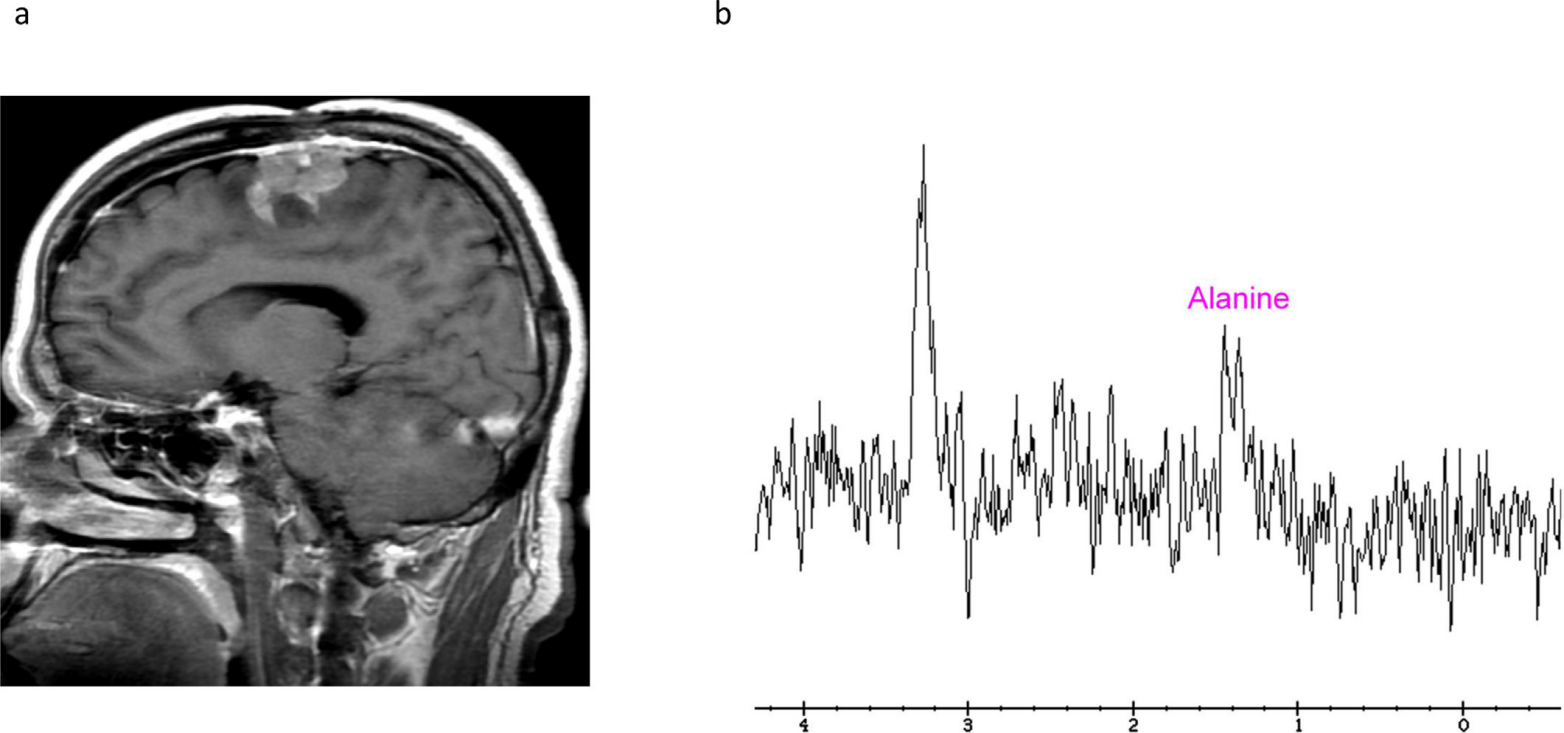
T2-FLAIR mismatch sign in IDH-mutant 1p19q intact astrocytoma**.** Axial T2-weighted (A), FLAIR (B), and contrast T1-weighted (C) images demonstrate T2-FLAIR mismatch with high T2 signal and low FLAIR signal in this non-enhancing IDH1 R132H-mutant low-grade astrocytoma centered in the insula and subinsula.

Although positive 2HG MRS is a highly specific marker for the presence of IDH mutation, it does not help with the next core classification, which requires determination of 1p19q status. Several studies have described the presence of the T2-FLAIR mismatch sign, characterized by hyperintense signal on *T*_2_-weighted sequences and hypointense signal on FLAIR sequences with a hyperintense peripheral rim, as a reliable and highly specific marker of IDH-mutant and 1p19q intact gliomas (astrocytomas).^40–43^ Figure 3. The mismatch sign is thought to reflect microcystic changes,^43^ and has been found only in lower grade gliomas.^41^ Although specificity is high, suggesting that the T2-FLAIR mismatch sign is a reliable indicator of IDH-mutant 1p19q intact astrocytomas, the sensitivity is variable and effect on clinical course is uncertain.^40,42^

### Radiomics

Radiomics is the high-throughput voxel-based quantitative analysis of medical images.^44^ Analysis of radiomic features can provide an alternative, noninvasive method that utilizes automatic data-characterization algorithms to extract features from radiographic images.^45,46^ IDH-mutant versus IDH-wildtype lower grade gliomas have shown differences in radiomic descriptors extracted from *T*_2_-weighted images. Radiomic features, including first order statistics (energy, entropy, mean, median, and root mean square), shape and size-based features (surface-to-volume ratio), textural features, and wavelet features were assessed and compared between IDH-mutant and IDH-wildtype lower grade gliomas. Analysis suggested the potential to distinguish between IDH-mutant and wildtype using radiomic signatures, with a total of 146 features found to be different. Further analysis found the radiomic features of surface-to-volume ratio and entropy to be associated with the same biological processes found to differ between IDH-mutant and wild-type lower grade gliomas, which includes cell polarity, cell adhesion, cell growth, and immune processes. Further research into the utilization of noninvasive imaging methods may aid to predict prognosis and treatment options without the need for invasive tissue sampling.^45^

### MRI spectroscopy to detect other oncometabolites

IDH-mutant gliomas can result in multiple metabolite aberrations other than the 2HG oncometabolite. Some of these aberrations are not unique to IDH-mutant gliomas, such as increased choline peak near 3.2 ppm, which sums contributions from multiple choline-containing metabolites, including free choline, phosphorylcholine, and glycerophosphorylcholine. Choline is increased in many tumors due to increased cell membrane synthesis and increased cellularity, which in IDH-mutant gliomas may be due to mutant IDH-mediated cellular proliferation.^21^ Gliomas may also demonstrate decreased creatine peak at 3.03 ppm, which sums contributions from creatine and phosphocreatine, GABA, lysine, and glutathione. Creatine is an intracellular energy marker that is often decreased in gliomas, particularly high-grade gliomas, although it may be relatively constant in other tumors.

Unlike IDH-wildtype gliomas, the IDH-mutant gliomas may also demonstrate decreased glutamate (Glu), myo-inositol (mI), glutathione (GSH), and glucose+taurine.^21,25^ The IDH1 mutation also influences glutamate metabolism, with expression of glutamate dehydrogenase 1 (GDH1) and glutamate dehydrogenase 2 (GDH2) mRNA significantly higher in IDH1-mutant gliomas than in IDH1-wildtype gliomas.^47^ IDH1-mutant gliomas also expressed decreased mRNA level of branched chain amino acid transaminase 1 (BCAT1). GDH catalyzes the conversion of glutamate to α-ketoglutarate and ammonia while reducing NAD(P)+ to NAD(P)H. On the other hand, BCAT1 functions to catalyze α-ketoglutarate to glutamate within the cytosol.^47,48^ Preclinical work has demonstrated the feasibility of real time *in vivo* imaging of 2HG formation from glutamate using hyperpolarized^14^C MRI.^49^ While further study is needed to fully elucidate the details of glutamate metabolism, the decrease in glutamate in IDH-mutant gliomas may represent the consumption of glutamate as a primary carbon source for the 2HG oncometabolite production, or an attempt to restore α-ketoglutarate lost through the further conversion by IDH-mutant enzyme to 2HG.^47,49^

Increased lipid and lactate inverted doublet peaks at 1.3 ppm are often found in glioblastoma and other high-grade malignancies. The lipid peak is attributed to microscopic cellular necrosis with cell membrane destruction releasing mobile fatty acyl moities. The lactate peak is attributed to anaerobic metabolism, although its presence is variable and does not consistently correlate with glioma grade. Radiation necrosis may also demonstrate increased lipid and lactate peaks, although those lesions often show minimal or are absent other normal metabolites. Lipid may also be increased in primary CNS lymphoma from the synthesis or release of fatty moities during cell turnover or differentiation, rather than usual tumoral necrosis, or from lymphocyte and leukocyte activation or transformation.^50^ Unfortunately, in contrast to the high fidelity of 2HG for IDH mutation, lactate may be increased in many other pathologic conditions, including ischemia and mitochondrial diseases.^51^

**Figure 4.**
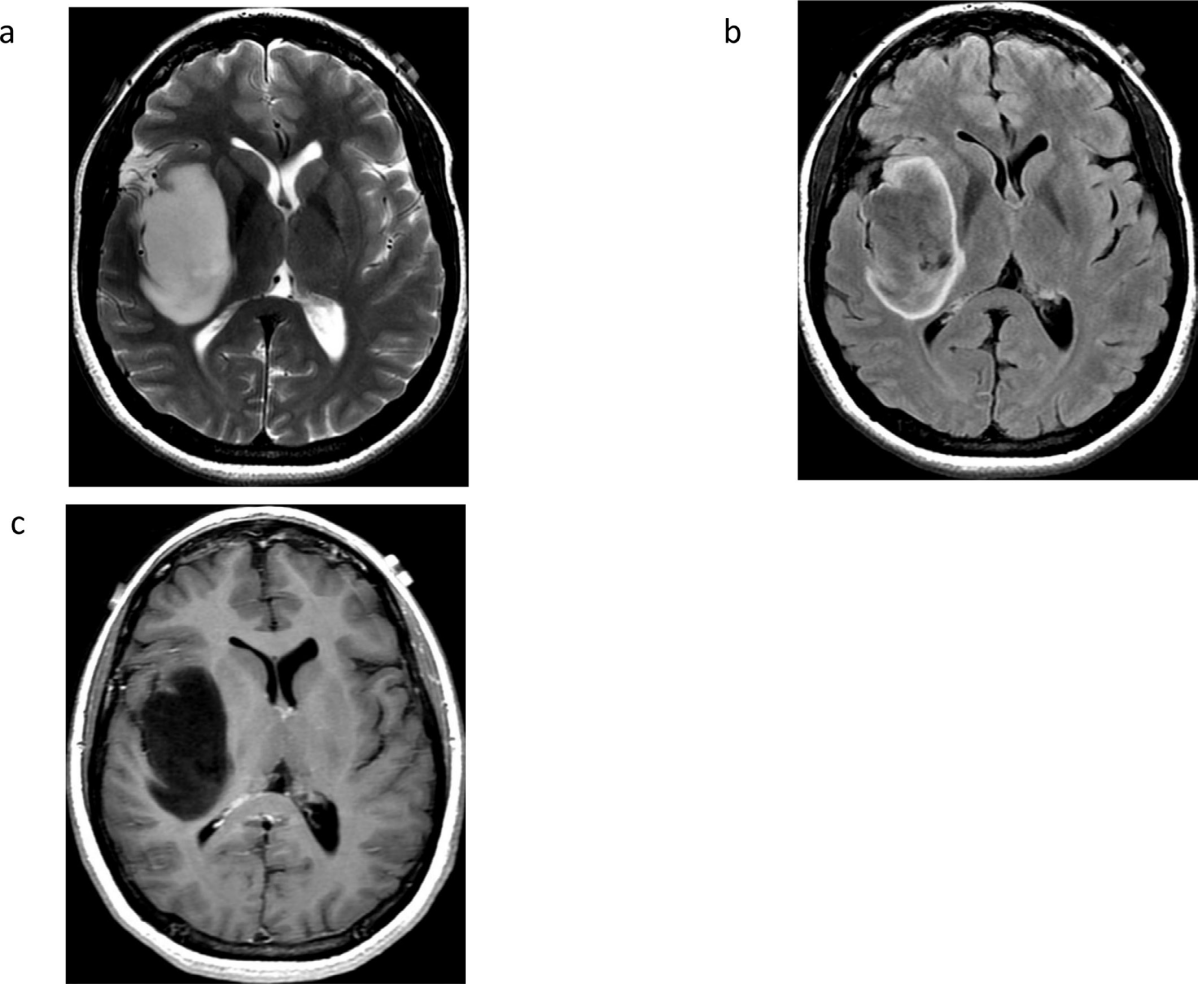
Increased alanine doublet in meningioma**.** Sagittal contrast T1-weighted image (A) shows a recurrent WHO Grade 1 meningioma. Spectroscopy with TE=144ms (B) reveals a typical alanine doublet at 1.48 ppm

An additional oncometabolite of interest is alanine, an amino acid with doublet peak at 1.48 ppm that inverts at intermediate TE due to J-coupling. The alanine is produced by pyruvate transamination in hypoxic tissues with nitrogen donation from glutamate. Increased alanine is considered specific to meningiomas and helpful in differentiating them from other tumors, although the sensitivity is quite variable. Figure 4. The increased alanine in atypical meningiomas, WHO Grade 2, may be driven by increased glutamate and glutamine precursors, as well as metabolic reprogramming to upregulate alanine as an alternative energy source to support tumor growth.^52^ Increased lactate and glycine may also occur in atypical meningiomas, WHO Grade 1. Interestingly, a distinct large peak at 3.8 ppm is also considered to be specific for meningioma, although the cause of the peak and underlying oncometabolite remains uncertain.^53,54^

## Further mutation classifications and their imaging indicators

### 1p19q codeletion in IDH-mutant glioma defines oligodendroglioma

Previous studies have shown lower grade gliomas with IDH mutation to have either 1p19q codeletion or a TP53 mutation.^16^ All tumors with a complete 1p19q codeletion, considered the hallmark of oligodendroglioma, also have a mutation in IDH1 or IDH2.^45,46^ The 1p19q codeletion is more common in IDH2-mutant tumors than in IDH1-mutant tumors (91% vs  48%, *p* < 0.001).^55^ The IDH-mutant and 1p19q codeleted status is diagnostic of oligodendroglioma even in the absence of any oligodendroglioma-looking tumor cells at microscopy. The 1p19q molecular alteration is a defining event caused by an unbalanced whole-arm translocation between chromosome 1 and chromosome 19, and has prognostic and predictive value as patients enjoy better overall survival and are more responsive to treatment with chemotherapy than comparable grade astrocytomas.^56–58^ The biologic and metabolic consequences of 1p19 codeletion, however, remain unclear although they do not induce an obvious oncometabolite like 2HG with IDH mutation. The 1p19q codeleted oligodendrogliomas have been described as having less immune cell infiltration and lower immune checkpoint gene expression, probably in part related to deletion of immune-related genes.

Amine chemical exchange saturation transfer (CEST) imaging has suggested less acidity in 1p19q codeleted gliomas than in 1p19q intact gliomas, although this was not corroborated by L-6–18F-fluoro-3,4-dihydroxyphenylalnine (18F-FDOPA) PET imaging.^59^ Amide proton transfer-weighted (APTw) imaging shows lower APTw signal in IDH-mutant gliomas than in IDH-wildtype gliomas.^60,61^ This APT signal change is attributed to increased amide proton concentration in tumors relative to mobile cellular proteins and contribution from alkaline intracellular pH, as well as lower APTw from the global downregulation of protein expression in the IDH-mutant gliomas.^62^

### Diffuse midline glioma, H3 K27M-altered

The 2021 WHO Classification described four pediatric high-grade gliomas, including the newly re-named “diffuse midline glioma, H3 K27-altered” instead of the older “H3 K27-mutant” term.^2^ Diffuse midline gliomas are commonly characterized by histone H3 lysine27-to-methionine mutations with K27M mutations in the histone H3 gene H3F3A, although other mechanisms, such as EZHIP protein overexpression, may also present as driving alterations. The H3 K27M mutation is also not exclusive to H3 K27M mutant diffuse midline gliomas, as it may occur in other tumors, including ependymomas, pilocytic astrocytomas, pediatric diffuse astrocytomas, and gangliogliomas. Therefore, as per the cIMPACT-NOW update 2,^63^ the H3 K27M mutation cannot solely define the cohort, unlike gliomas defined by IDH status or oligodendrogliomas defined by 1p19q codeleted status. The diffuse growth pattern classically occurs in a midline location.^2^ These tumors can only be diagnosed when all the terms in the name “diffuse midline glioma, H3 K27M-altered” are present: diffuse (infiltrating), midline (thalamus, brainstem, spinal cord), glioma, and H3 K27-specific neuroglial mutations. The H3 K27M changes lead to global decreases in H3 K27me3 through faulty PRC2 interactions and H3 K27me3 spreading to silence tumor suppressor genes.^63^ The H3 K27M alterations are often accompanied by oncogenic aberrations in pathways involved in cell damage repair, DNA damage repair, and receptor tyrosine kinase signaling. Because of these widespread metabolic and epigenetic alterations, H3 3K27M alterations can be considered mutually exclusive from IDH mutations. Since they affect closely integrated pathways, having both mutations would induce synthetic lethality.^64^

**Figure 5.**
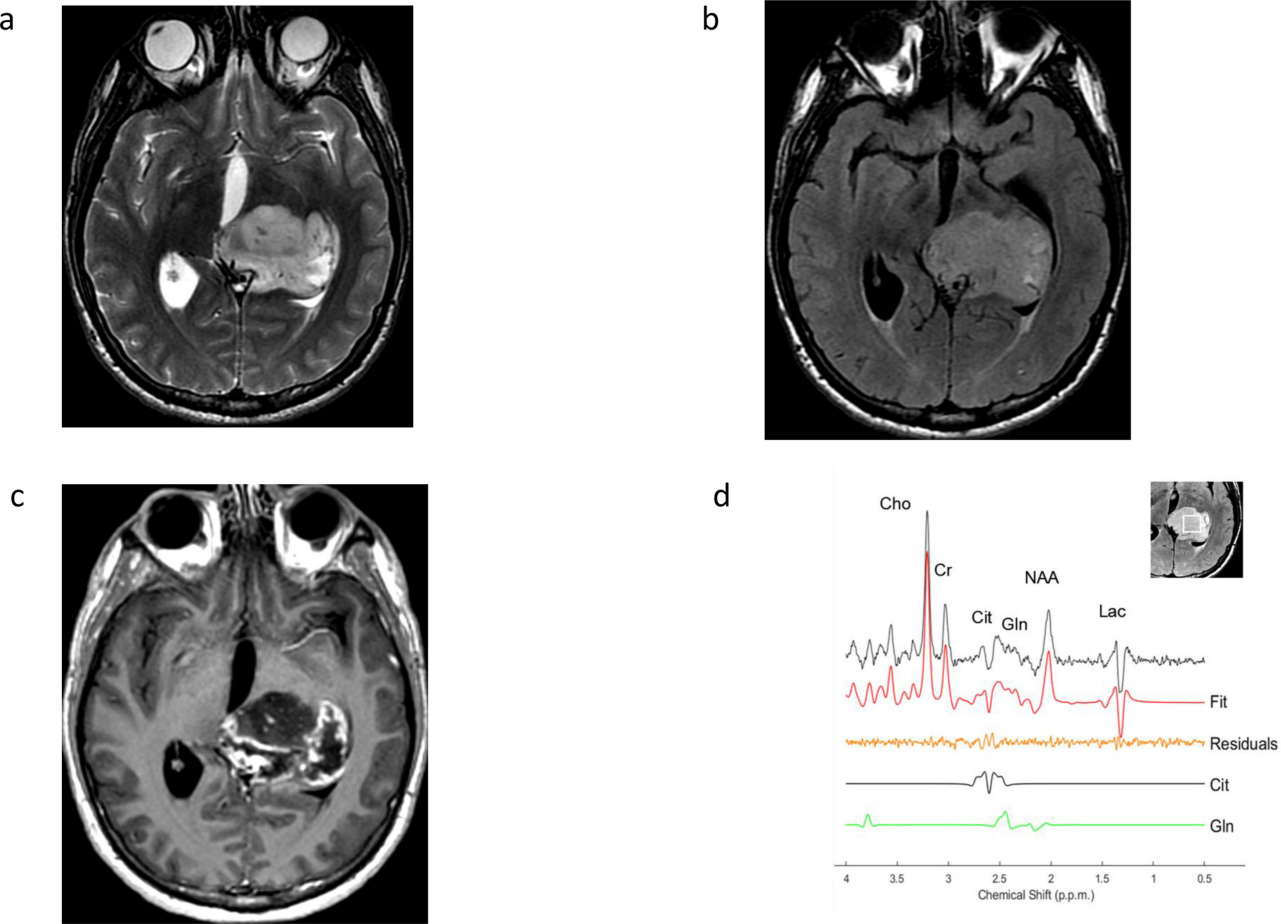
Increased glutamine and citrate in H3 K27M-altered diffuse midline glioma. Axial T2-weighted (A), FLAIR (B), and contrast T1-weighted (C) images (D) in a patient with a heterogeneous H3 K27M-altered diffuse midline glioma, WHO Grade 4 in the thalamus. Spectroscopy (D) shows characteristic increased glutamine (7.7mM, 14% CRLB) at 2.2–2.4 ppm and increased citrate (3.9 mM, 8% CRLB) at 2.6 ppm, in addition to typical tumor increase in choline, decrease in NAA, and increase in lipid/lactate.

Standard MRI features that suggest H3 K27M mutations in diffuse midline gliomas include increased enhancement, increased enhancement thickness, less edema, better defined nonenhancing margins, and less cortical invasion.^65^ With advanced MRI, the H3 K27M mutant gliomas demonstrate higher relative cerebral blood volume and lower peritumoral apparent diffusion coefficient (ADC) at perfusion and diffusion imaging,^66^ as well as increased citrate and increased glutamate at MRS due to increased glycolysis, glutaminolysis, and TCA cycle metabolism.^64^ Figure 5. Machine learning-based radiomic analysis may also be helpful to predict H3 K27M mutation status.^67^ The perseverance of these imaging markers examining “H3 K27M-mutant” gliomas, however, is uncertain given the more recent inclusion of “H3 K27M-altered” gliomas.

## Conclusion

Discoveries of the genomic alterations driving cancer development and growth have led to advances in tumor classification, prognosis, and increasingly targeted treatment options. These advances have improved our understanding of cancer biology, however efforts to fully elucidate the oncogenic roles of the oncometabolite products is ongoing. Some critical oncometabolites are amenable to imaging characterization and quantification, promising opportunities for new insights into cancer behavior.
